# A Parallel Tracking of Salivary and Gut Microbiota Profiles Can Reveal Maturation and Interplay of Early Life Microbial Communities in Healthy Infants

**DOI:** 10.3390/microorganisms10020468

**Published:** 2022-02-18

**Authors:** Sofia Reddel, Giuseppe Rubens Pascucci, Silvia Foligno, Federica Del Chierico, Pamela Vernocchi, Alessandra Marzullo, Maria Grazia Pattumelli, Paolo Palma, Guglielmo Salvatori, Lorenza Putignani

**Affiliations:** 1 Multimodal Laboratory Medicine Research Area, Unit of Human Microbiome, Bambino Gesù Children’s Hospital, IRCCS, 00165 Rome, Italy; sofia.reddel@opbg.net (S.R.); federica.delchierico@opbg.net (F.D.C.); pamela.vernocchi@opbg.net (P.V.); 2 Research Unit of Clinical Immunology and Vaccinology, Academic Department of Pediatrics (DPUO), Bambino Gesù Children’s Hospital, IRCCS, 00165 Rome, Italy; grubens.pascucci@opbg.net (G.R.P.); paolo.palma@opbg.net (P.P.); 3 Chair of Pediatrics, Department of Systems Medicine, University of Rome “Tor Vergata”, 00133 Rome, Italy; 4 Neonatology Department, Bambino Gesù Children’s Hospital, IRCCS, 00165 Rome, Italy; silvia.foligno@opbg.net (S.F.); marzullo.alessandra@gmail.com (A.M.); 5 Unit of Pediatric and Neonatal Intensive Care Unit, Belcolle Hospital, 01100 Viterbo, Italy; mpattumelli@gmail.com; 6 Unit of Microbiology and Diagnostic Immunology, Unit of Microbiomics and Multimodal Laboratory Medicine Research Area, Unit of Human Microbiome, Department of Diagnostic and Laboratory Medicine, Bambino Gesù Children’s Hospital, IRCCS, 00165 Rome, Italy

**Keywords:** oral microbiota, gut microbiota, core microbiota, networks analysis, ecological niche, newborns

## Abstract

In this study, the onset and shaping of the salivary and gut microbiota in healthy newborns during the first period of life has been followed, evaluating the impact of salivary microbiota on the development of early fecal microbial communities. The microbiota of 80 salivary and 82 fecal samples that were collected from healthy newborns in the first six months of life, was investigated by 16S rRNA amplicon profiling. The microbial relationship within and between the saliva and gut ecosystems was determined by correlation heatmaps and co-occurrence networks. *Streptococcus* and *Staphylococcus* appeared as early commensals in the salivary microbiota, dominating this ecosystem through the time, while *Fusobacterium*, *Prevotella*, *Porphyromonas*, *Granulicatella*, and *Veillonella* were late colonizers. Enterobacteriaceae, *Staphylococcus* and *Streptococcus* were gut pioneers, followed by the anaerobic *Bifidobacterium*, *Veillonella*, *Eggerthella*, and *Bacteroides*. *Streptococcus*, *Staphylococcus*, and *Veillonella* were shared by the gut and saliva ecosystems. The saliva and gut microbiota seem to evolve independently, driven by local adaptation strategies, except for the oral *Streptococcus* and *Veillonella* that are involved in gut microbiota development as seeding species. This study offers a piece of knowledge on how the oral microbiota may affect the gut microbiota in healthy newborns, shedding light onto new microbial targets for the development of therapies for early life intestinal dysbiosis.

## 1. Introduction

It is now largely recognized that microbial colonization in the human intestine begins immediately at birth [1,2,3,4] or even before delivery, during intrauterine life [5,6,7,8,9,10]. The infant’s gut microbiota is a highly dynamic community that is progressively and continuously modulated during life, with delivery and feeding representing the most relevant factors driving its onset and shaping [1,11,12,13].

Alterations of the gut microbiota, intended as dysbiosis, during neonatal life and infancy have been associated with paediatric disorders and with the establishment of several diseases during adult life [14,15]. Consequently, understanding the gut microbiota colonization dynamics in early life is not only intriguing in terms of microbial ecology, but also it may give novel insights into the relationship between the microbiome traits and disease triggering [16].

Contrary to the enormous number of studies that are available on the development of an infants’ gut microbiota [4,17,18,19,20,21], information on infant oral microbiota is mainly focused on mother–child interactions [22,23,24,25]. However, the progressive composition of infant oral microbiota is still limited. Only recently, a longitudinal study was conducted to characterize the oral microbiota in infants followed from three months to seven years of age [26]. The oral microbiota compositional patterns changed through the first two ages of life, starting with the “early colonizers”, including *Streptococcus*, *Veillonella*, and *Lactobacillus* spp., and ending with the “late colonizers”, such as *Actinomyces*, *Porphyromonas*, *Abiotrophia*, and *Neisseria* [26].

In this study, we addressed the establishment and development of the salivary and gut microbiota in the first six months of life in healthy newborns. Moreover, by correlation analysis and co-occurrence network approaches, the ecological progression of the two ecosystems during a time course was investigated to highlight the impact, through transmission, of the salivary microbiota on the development of early fecal microbial communities.

## 2. Materials and Methods

### 2.1. Study Subjects and Sample Collection

From April to October 2015, at the Unit of Gynecology and Obstetrics of Fatebenefratelli Hospital of Rome in Italy, 116 healthy newborns were consecutively enrolled in this observational cross-sectional study.

The inclusion criteria regarded healthy newborns that were vaginally-delivered at term (37–42 gestational weeks) with a normal birth weight (2.5–4.5 kg), breastfed, and with an age range of 0–180 days. The exclusion criteria included caesarean section delivery, antibiotic intake, and acute or chronic gastrointestinal diseases that were registered in the 30 days before the starting point of both the stool and saliva collection and also during the entire time course.

The Hospital Ethics Committees approved the study (“Protocol 784_OPBG_2014”), and parents signed the informed consent for this study. The saliva samples were collected by gently swabbing the infants’ cheeks with a sterile cotton swab at 7, 15, 30, 90, and 180 days of time life. The saliva samples at birth were not collected to avoid the disturbance of mother and neonate privacy during the baby’s first day of life, causing a potential stressful situation that might compromise the breastfeeding. The stool samples were collected at birth (meconium) and 7, 15, 30, 90, and 180 days from infant diapers (Table 1). To increase the study feasibility and reduce the risk of dropout, we enrolled different babies at each time point.

All the samples were collected during routine visits at Bambino Gesù Children’s Hospital and Research Institute of Rome, except for meconium samples that were collected at birth at the Unit of Gynecology and Obstetrics of Fatebenefratelli Hospital, Rome, Italy.

The samples were daily stored at 4 °C at the clinical unit and then, as soon as possible, they were transported at a controlled temperature and were stored long-term at −80 °C, at the BBMRI Biobank Microbiome node of the Human Microbiome Unit of Bambino Gesù Children Hospital, until laboratory processing.

### 2.2. Fecal and Salivary Microbiota Profiles

From the fecal samples, DNA was extracted by QIAmp Fast DNA Stool mini kit (Qiagen, Hilden, Germany), while from the saliva ones, DNA was extracted by the automatic extractor biorobot EZ1, using EZ1 DNA Tissue Kit, following the manufacturer’s instructions (Qiagen, Hilden, Germany).

The variable region V3–V4 of the 16S rRNA locus was amplified using primers that are described in the 16S Metagenomic Sequencing Library Preparation protocol (Illumina, San Diego, CA, USA). The first PCR mix was prepared with the Fast Start Hifi Taq (Roche Diagnostics GmbH, Mannheim, Germany) and the reaction set up as follows: initial denaturation at 95 °C for 3 min, 32 cycles of denaturation at 95 °C for 30 s, annealing at 55 °C for 30 s, extension at 72 °C for 30 s, and final extension step at 72 °C for 5 min. The DNA amplicons were purified with 20 µL of KAPA Pure Beads (Roche Diagnostics, Germany). The second PCR step was performed by Nextera indexes (Illumina, USA) to obtain a unique combination of barcoded sequences. In each PCR step, the negative (no-template) and positive (ZymoBIOMICS™ Microbial Community DNA Standard, Zymo Research, Irvin, CA, USA) controls were used to monitor and exclude eventual external and internal contaminants. The final library was purified with 50 μL of KAPA Pure Beads, quantified using Quant-iT™ PicoGreen^®^ dsDNA Assay Kit (Thermo Fisher Scientific, Waltham, MA, USA), and diluted in equimolar concentrations (4 nM). Subsequently, the samples were pooled, denatured, diluted at 7 pM, and sequenced on an Illumina MiSeqTM platform (Illumina, USA).

### 2.3. Biocomputational and Statistical Analyses

The Timmomatic pipeline was used to preprocess the Illumina Miseq paired-end reads in terms of quality and length [27]. The ChimeraSlayer algorithm was used to check the chimera sequences [28]. By QIIME software, the sequences were organized into operational taxonomic units (OTUs) with a 97% of clustering threshold of pairwise identity by closed-reference OTU picking process. The PyNAST v.0.1 program was used to carry out a multiple sequence alignment against the Greengenes 13_08 database with a 97% similarity for the bacterial sequences [29]. The OTUs multiple sequence alignment was used to build a phylogenetic tree [30]. Only for ecological analyses, the sequences were subsampled to the smallest number of sequences [31]. Shannon and Chao1 indices, unweighted UniFrac matrix, and PERMANOVA test (9999 permutations) were performed by Phyloseq and vegan packages of R software [32]. For the following analyses, the OTU table was obtained from the data without any previous rarefaction procedures and normalized by the total sum scaling method. The saliva and fecal OTU tables were filtered to retain OTUs that were present at least 20% of all the samples. Significant changes in the OTUs relative abundances at phylum and genus levels were assessed by the Kruskal–Wallis test, corrected for multiple tests (*p*FDR < 0.05). Only taxa with a relative abundance that was higher than 0.01 (0.01%) were considered for the computational analysis at the genus level. The core microbiota of the saliva and stool samples was detected by retaining those genera that were present in at least 50% of the samples of the corresponding group by compute_core_microbiome.py script of QIIME.

Heatmaps and co-occurrence networks were obtained from all the time points that were included in this study. Pearson’s correlation analysis was used to find significant associations between the relative abundance values at the phylum and genus taxonomic levels. Only statistically significant correlations (*p*FDR < 0.05) were shown in graphical representations. Statistical analyses were performed using R (version 3.6.2).

Co-occurrence networks were produced with Cytoscape 3.7.2 version and network analysis was performed with Network Analyzer plug-in.

## 3. Results

### 3.1. Microbiota Profiling

At the end of enrolment period, we enrolled 116 healthy newborns fulfilling the inclusion criteria, of which 34 were excluded from the study due to additional medication administration and a lack of signed informed consent. Finally, 82 fecal and 80 saliva samples were collected from 82 infants through a time course that is described in Table 1.

From the stool samples, a total of 4,774,402 sequencing reads were obtained, with a minimum value of 6042, a maximum value of 175,894 and a mean value of 50,791 sequences per sample. From saliva samples, a total of 1,710,409 sequencing reads were obtained, with a minimum value of 3079, a maximum value of 35,808, and a mean value of 17,816 sequences per sample.

To evaluate the effect of confusion factors on stool and salivary microbiota distribution, we first performed a principal component analysis (PCA) on OTUs abundances using a “gender” variable. We observed the gender did not influence the distribution of the OTUs abundances neither in the stool nor in the salivary microbiota (Supplementary Figure S1). Moreover, we used the variable age to group and analyze our sample to investigate the development of the salivary and fecal microbiota during the first six months of baby’s life.

**Figure 1:**
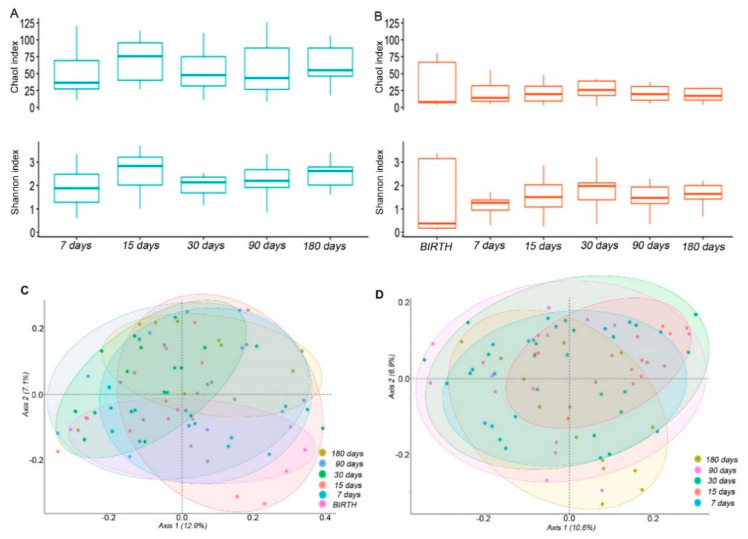
Ecology of the salivary and intestinal microbiota. (**A**,**B**) box plots of the Chao1 and Shannon indexes of the saliva (**A**) and stool (**B**) samples. Boxes represent the minimum and maximum values, median, 25th, and 75th percentiles of indexes at the indicated time points. (**C**,**D**) Principal coordinates analysis (PCoA) plots of the unweighted UniFrac matrix of all the time-points for the saliva (**C**) and stool (**D**) samples. The shape of the symbols refers to the microbiota matrix, while the colour indicates the time point. The plots show the percentage of variance that is explained in the first two axes.

Following ecological analyses of the salivary and intestinal microbiota, in the saliva samples, an increase of α-diversity was observed since the 7 days’ time point, with a peak at 15 days for both the Shannon and Chao1 indexes (Figure 1A). Particularly, Shannon and Chao1 were significantly higher at time 15 compared to time 7 (*p* = 0.048 and *p* = 0.025, respectively), while only the Shannon resulted in significantly higher α-diversity at day 180 versus day 7 (*p* = 0.037, Figure 1A).

The α-diversity measures in the fecal samples followed an increasing tendency during the 7–180 days’ time course (Figure 1B), similar to the saliva samples even with a lower complexity. The highest mean value for the Shannon and Chao1 indexes was observed at 30 days. At day 30 and 180 there was a significant increase of the Shannon index compared to day 7 (*p* = 0.031 and *p* = 0.019, respectively).

However, the α-diversity was significantly higher in the saliva compared to the stool samples at each newborns’ time point, except for the Shannon value that was measured at 30 days (Supplementary Table S1).

The compositional dissimilarity between the time-points in terms of phylogenetic relatedness was evaluated by β-diversity. For both the saliva and fecal samples, the OTUs distribution differences at the different time-points were statistically significant as assessed by the PERMANOVA test (*p* = 0.001 and *p* = 0.032, respectively). The separation in the first axis was stronger for the fecal than the saliva samples (Figure 1C,D).

The microbiota composition across the saliva and feces body matrices showed a significantly different structure, as assessed by the PERMANOVA analysis (*p* = 0.0001; Supplementary Figure S2).

### 3.2. Salivary Microbiota Profiling

**Figure 2:**
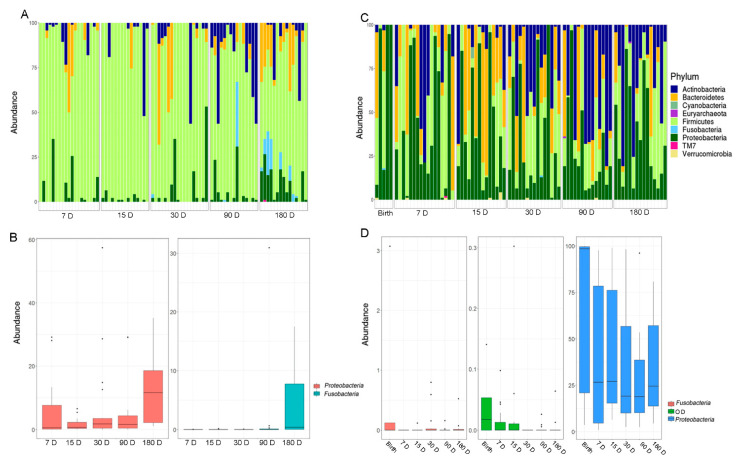
(**A**) The OTUs distribution of the salivary microbiota at the phylum level. (**B**) The histograms refer to the Kruskal–Wallis test-based phyla distribution with a *p*FDR < 0.05 in the salivary samples. (**C**) The OTUs distribution of the gut microbiota at the phylum level. The bar graphs represent the average distribution of the OTUs. (**D**) The histograms refer to the Kruskal–Wallis test-based phyla distribution with a *p*FDR < 0.05 in stool samples. D, days.

Firmicutes largely dominated the salivary microbiota during all the time courses. At 90 days, there was a strong increase of Actinobacteria, but these results changed at 180 days, for most of the samples, towards an increase of Proteobacteria (*p*FDR < 0.05), Fusobacteria (*p*FDR < 0.05), and Bacteroidetes (Figure 2A,B).

**Figure 3:**
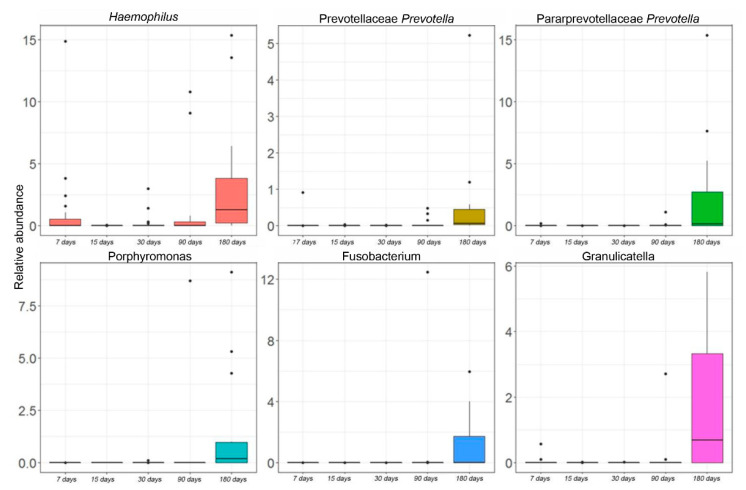
Kruskal–Wallis test-based OTU distribution of the salivary microbiota. The bar graphs represent the average distribution of the OTUs at the genus level in salivary microbiota during the first six months of infant’s age. Only statistically significant OTUs with *p*FDR < 0.05 are shown.

At the genus level, statistically significant differences were found during the time course for six OTUs. By this analysis, *Haemophilus* (Proteobacteria), *Porphyromonas* (Bacteroidetes), *Prevotella* (Bacteroidetes), *Fusobacterium* (Fusobacteria), and *Granulicatella* (Firmicutes) were increased at the last point of the time course (Figure 3).

From an analysis of the OTUs that were shared between all-time points, we were able to identify a temporal core microbiota in the saliva samples (Supplementary Table S2). The most prevalent OTUs (≥1% abundance) were *Rothia*, *Staphylococcus*, *Streptococcus*, Gemellaceae_g, and *Veillonella. Staphylococcus* and *Streptococcus* were the predominant genera of the infant salivary core microbiota during the first six months of age. In particular, *Streptococcus* presented a remarkable decrease of its relative abundance from 75.8% to 66.9% at 180 days’ time point (Supplementary Table S2).

### 3.3. Gut Microbiota Profiling

The fecal microbiota was characterized by a more homogenous distribution of phyla compared to the salivary one. The entire time course showed a high percentage of Proteobacteria with a relative abundance distribution that varied significantly over time (*p*FDR < 0.05) (Figure 2C,D). On day 15, there was a substantial increase of Bacteroidetes with a gradual transition towards a rise in Actinobacteria until the last time point. The phyla Proteobacteria, Fusobacteria, and OD1 showed a decreasing trend from birth over time (*p*FDR < 0.05).

**Figure 4:**
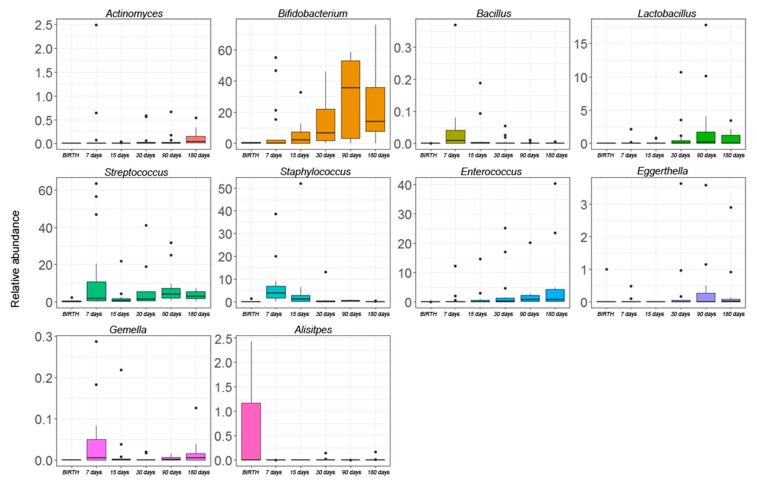
The Kruskal–Wallis test-based OTU distribution of the gut microbiota. The bar graphs represent the average distribution of the OTUs at the genus level in the gut microbiota during the first six months of infant’s age. Only statistically significant OTUs with *p*FDR < 0.05 are shown.

At the genus level, the Kruskal–Wallis test revealed significant differences for 10 OTUs. *Actinomyces*, *Bifidobacterium*, and *Enterococcus* followed an increasing trend during the entire time course. In particular, *Bifidobacterium* appeared at day 7, significantly increased over time, until a blow up at day 90. *Eggerthella* and *Lactobacillus* increased until day 90 but started to decrease from day 180. *Bacillus* and *Staphylococcus* showed a peak of abundance a day 7 but decreased during the time course. *Streptococcus* and *Gemella* showed a constant increase in their relative abundances starting from day 15. *Alistipes* had high levels of abundance at birth, but during the next time-points, its levels were nearly zero (Figure 4).

An analysis of OTUs that were shared from birth to the first six months of age of our infant cohort revealed the presence of a gut core microbiota that was composed of 14 OTUs (Supplementary Table S2). Particularly, Enterobacteriaceae_g predominated in the meconium samples (>90% of abundance) but decreased at day seven, leaving space for other genera, including the most abundant *Bifidobacterium*, *Bacteroides*, *Staphylococcus*, *Streptococcus*, and *Veillonella* as represented in Supplementary Table S3.

### 3.4. OTUs at Genus Level Shared between Gut and Salivary Microbiota

We evaluated which OTUs were shared between the fecal and salivary microbiota during the time course. A deep analysis of the shared OTUs between the two ecosystems revealed that an average number of seven OTUs were present at each time point. In particular, *Staphylococcus*, *Streptococcus*, and *Veillonella* were shared during all points of the time course (Supplementary Table S4). The abundances’ trend over time of *Staphylococcus*, *Streptococcus*, and *Veillonella* was then analysed (Supplementary Figure S3A,B). *Staphylococcus* and *Streptococcus* were dominant and persistent colonizers in the saliva samples, while they showed a lower abundance in the fecal samples. Mainly, *Staphylococcus* had higher levels during the first days of life and decreased during the time, while *Streptococcus* showed a different trend in both the saliva and gut microbiota. In saliva, *Streptococcus* showed a decreasing trend during the last points of the time course, while in the gut microbiota a constant increase was exhibited after day 15. *Veillonella* increased over time both in the salivary and gut microbiota, showing higher levels in the saliva samples.

### 3.5. OTUs Correlations and Network Analysis in the Salivary Microbiota

**Figure 5:**
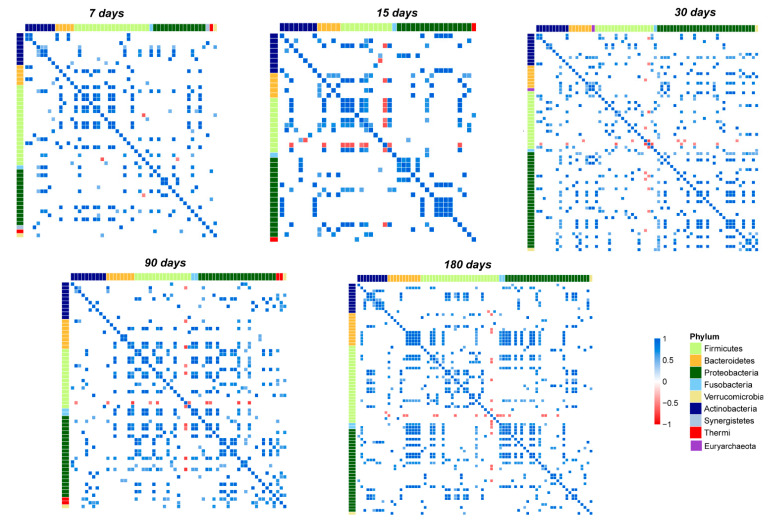
Pearson’s correlation heatmaps of salivary OTUs. Panels that are labeled as 7, 15, 30, 90, and 180 days show the correlation (colored squares) heatmaps between the OTUs. Pearson’s correlations with *p*FDR < 0.05 are shown. The color scale represents the correlation level: red, negative correlation values; blue, positive correlation values. The OTUs are colored according to the phylum level taxonomy.

During the evolution of the salivary microbiota, we observed an increase in the number of total OTUs correlations. This increase is particularly evident in the transition from day 15 to 30. In contrast, after day 90, we observed that correlation networks around the OTUs belonging to Firmicutes, Bacteroidetes, and Proteobacteria phyla started to converge into tightly condensed agglomerates (Figure 5).

**Figure 6:**
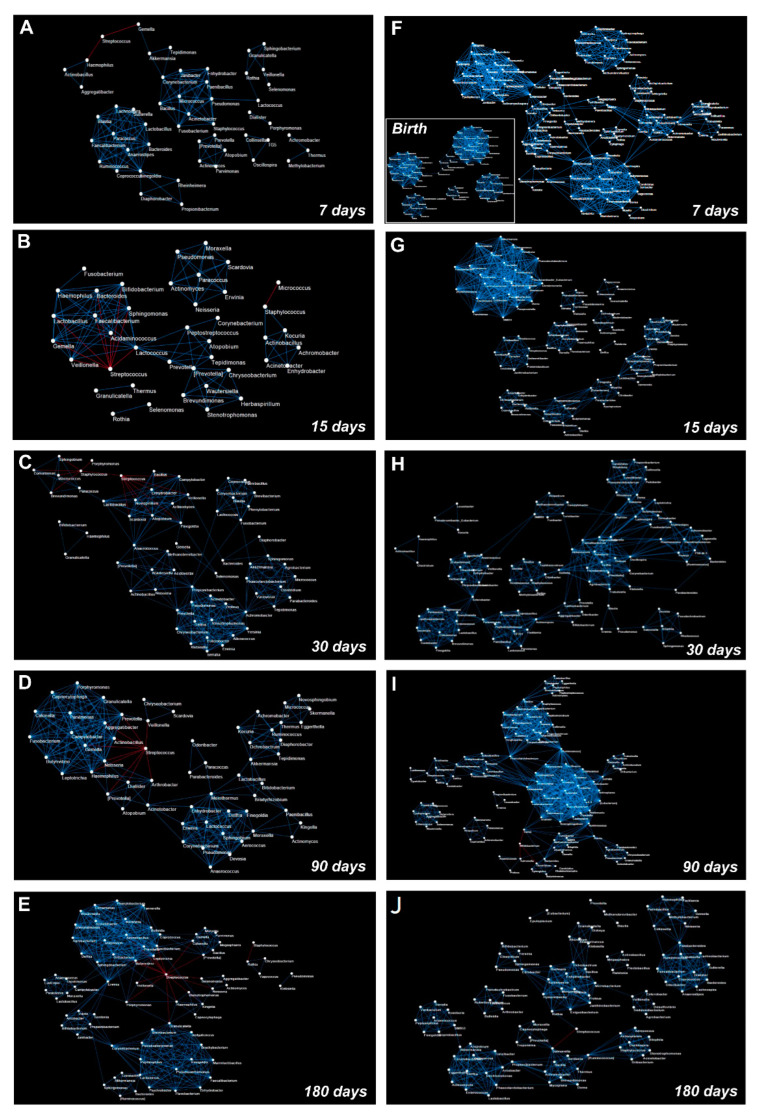
Graphical representation of the OTUs co-occurrence networks. The panels represent OTUs co-occurrence networks of saliva (**A**–**E**) and stool (**F**–**J**) samples that were analyzed at days 7, 15, 30, 90, and 180 following birth, respectively. The co-occurrence network that was obtained from meconium samples is represented as an inset within panel (**F**). The blue line indicates a positive correlation and a red line indicates a negative correlation. A Pearson’s test was used to evaluate the correlation amongst the OTUs (statistical significance was assessed with *p*FDR < 0.05).

Focusing on the most abundant OTUs in the salivary microbiota, we noted that *Streptococcus* established only negative correlations (Figure 5 and Figure 6A–E).

Indeed, on day seven, *Streptococcus* negatively correlated only with *Haemophilus* and *Gemella*. During the entire time course, its negative correlations increased until day 180, when it negatively correlated with *Veillonella*, *Haemophilus*, *Granulicatella*, *Prevotella*, *Porphyromonas*, and *Fusobacterium.*

*Staphylococcus* is the second most abundant out and showed negative correlations only at day 30 with *Streptococcus*, *Porphyromonas*, *Sphingobium*, *Brevundimonas*, *Comamonas*, and *Macrococcus*. Starting from day 90 its correlations decreased, and it maintained a negative correlation only with *Rothia* (Figure 5 and Figure 6C–E).

*Gemella* and *Veillonella* presented several positive correlations during the entire time course; none of these established stable relationships over time, with the only exception of a strong negative correlation with *Streptococcus* (Figure 5 and Figure 6A–E). *Rothia* presented no significant correlations at the point of its maximum abundance (day 90), showing a negative correlation with *Staphylococcus* during the last time points of the study (Figure 5 and Figure 6D,E). Network analysis showed that the highest number of OTUs and their relative positive correlations condensed into two large and four small clusters at day 180 (Figure 6A–E). These groups seem to evolve independently from each other, but some of their members, such as *Haemophilus*, *Granulicatella*, *Porphyromonas*, and *Fusobacterium*, established an interconnection through a negative correlation with *Streptococcus*.

### 3.6. OTUs Correlations and Network Analysis in the Gut Microbiota

**Figure 7:**
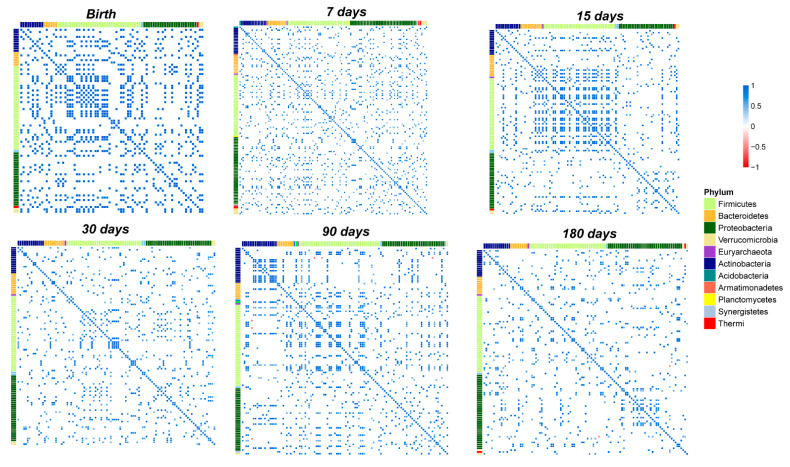
Pearson’s correlation heatmaps of fecal OTUs. Panels that are labelled as Birth, days 7, 15, 30, 90, and 180 show the correlation (coloured squares) heatmaps between the OTUs. Pearson’s correlations with *p*FDR < 0.05 are presented. The colour scale represents the correlation level of each variable: red, negative correlation values; blue, positive correlation values. The OTUs are coloured according to the phylum level taxonomy.

Based on the heatmap correlation analysis, we observed that the number of total correlations was higher compared to those that were established in the salivary microbiota (Figure 7).

Moreover, network analysis revealed a higher clusterization coefficient in the gut microbiota compared to the salivary microbiota (Supplementary Table S5).

Notably, from birth to day seven of life there was a strong increase in the number of total correlations (Figure 6F and Figure 7).

Furthermore, only positive correlations between the OTUs were detected at days 7, 15, and 30 (Figure 6F–H and Figure 7).

Focusing on the correlations that were established by the OTUs that we speculated were transferred from the saliva to the stool and on the correlations by the 10 OTUs which significantly changed during the time course of the gut microbiota maturation (Figure 4 and Supplementary Table S4), on day seven, *Streptococcus* established positive correlations with *Prevotella*, *Bacillus*, *Enterococcus*, *Haemophilus*, *Coprococcus*, *Dorea*, *Faecalibacterium*, and *Lactococcus.* On day 15, *Bifidobacterium* positively correlated with *Staphylococcus*, *Bacillus*, *Haemophilus*, and *Gemella*. Another positive correlation was observed between *Bacillus* and *Streptococcus* (Figure 6G and Figure 7).

On day 30, *Bifidobacterium* changed its positive correlations and interacted with *Actinomyces* (Figure 6H and Figure 7). Moreover, *Actinomyces* was part of an interaction network that involved *Lactobacillus* and *Enterococcus* (Figure 6H).

On day 90, negative correlations were detected for *Bifidobacterium* and *Dorea*, *Clostridium*, and *Salmonella*. Moreover, *Bacillus* positively correlated with a cluster of nodes in which *Actinomyces*, *Eggerthella*, and *Lactobacillus* were present (Figure 6I and Figure 7). At day 180, *Actinomyces*, *Eggerthella*, and *Lactobacillus* continued to be connected by positive correlations and positively cooperated with *Enterococcus* within the same cluster of nodes (Figure 6L and Figure 7).

### 3.7. OTUs Correlations and Co-Occurrence Network Analysis between Salivary and Gut Microbiota

**Figure 8:**
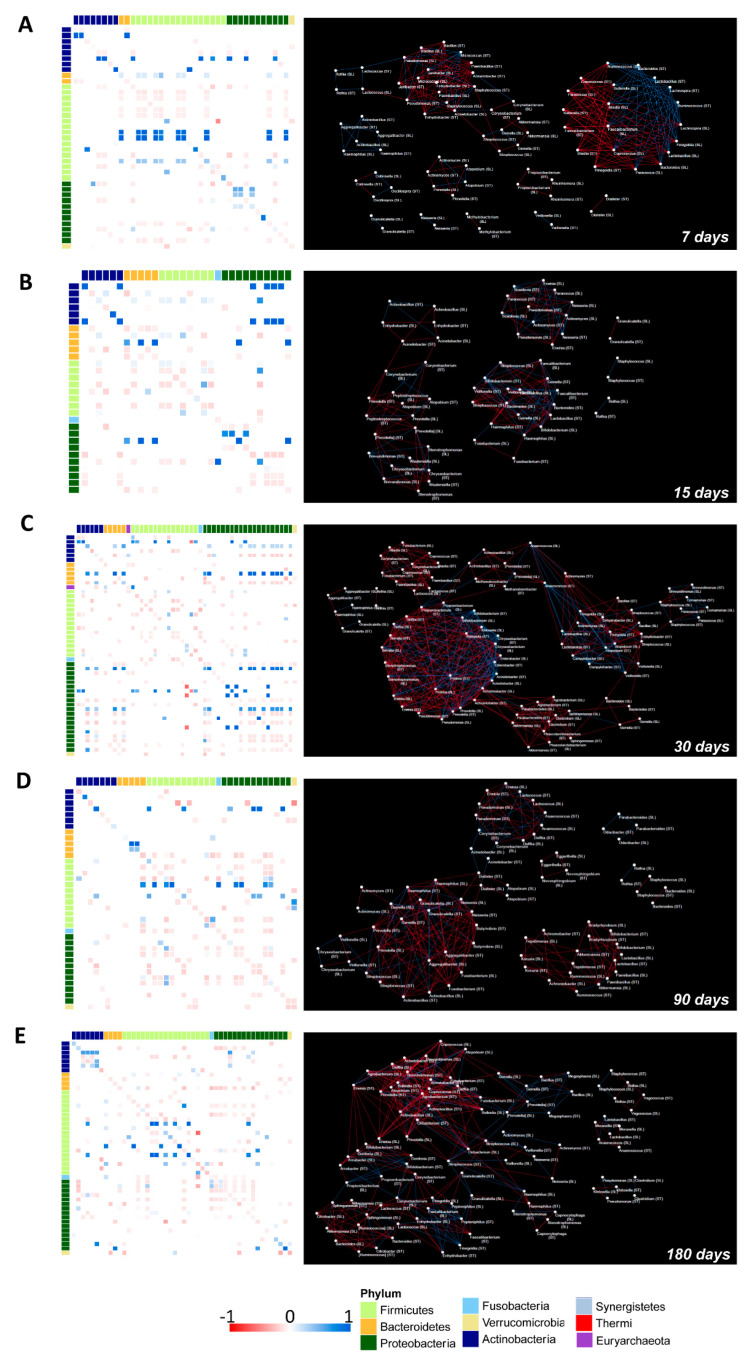
Correlation heatmaps and the OTUs co-occurrence networks of the saliva versus the stool microbiota from 7 to 180 days following birth. On the left side, Pearson’s correlation heatmaps between saliva versus stool OTUs. Panels (**A**–**E**) show the correlation (colored squares) heatmaps between the saliva versus the stool OTUs at days 7, 15, 30, 90, and 180. Pearson’s correlations with *p*FDR < 0.05 are presented. The color scale represents the correlation level of each variable: red, negative correlation values; blue, positive correlation values. The OTUs are colored according to the phylum taxonomy. OTUs co-occurrence networks are represented on the right side of the figure, calculated on heatmap correlations. The blue line indicates a positive correlation and the red line indicates a negative correlation.

Co-occurrence network analysis was used to study the relationship between the saliva and gut microbial communities (Figure 8). For this analysis, we focused on the OTUs that were shared between the salivary and gut microbiota (Supplementary Table S4).

Remarkably, on day seven we observed positive correlations between: *Staphylococcus* (saliva) versus *Staphylococcus* (stool), *Rothia* (saliva) versus *Rothia* (stool), *Veillonella* (saliva) versus *Veillonella* (stool), and *Gemella* (saliva) versus *Gemella* (stool). Interestingly, from the network analysis we visualized a cluster of interactions that were established by *Staphylococcus* (saliva) with several members of Actinobacteria, Proteobacteria, and Firmicutes phyla (Figure 8A).

*Streptococcus* (saliva) generated a negative correlation with *Gemella* (saliva), *Gemella* (stool), and *Streptococcus* (stool) (Figure 8A).

At seven days, a dense hub of positive and negative interactions was established between the OTUs belonging to Proteobacteria and Firmicutes that were characterized in both ecosystems. At 15 days, an isolated weak positive correlation between *Staphylococcus* (saliva) and *Staphylococcus* (stool) occurred while correlations between *Streptococcus* (saliva vs. stool) and *Gemella* (saliva vs. stool) changed into negative. Network analysis showed that *Streptococcus*, *Gemella*, and *Veillonella* that were characterized in the stool and saliva samples were part of a dense cluster of nodes (Figure 8B).

On day 30, *Staphylococcus* (saliva) still positively correlated with *Staphylococcus* and *Streptococcus* (stool) but established also strong negative correlations. *Streptococcus* (saliva) presented negative correlations with *Atopobium* (stool), *Campylobacter* (stool), and *Veillonella* (stool). From network analysis, a dense hub of positive and negative correlations involved several OTUs that were characterized in both the saliva and gut microbiota, the great majority belonging to the Proteobacteria phylum (Figure 8C).

On day 90, *Streptococcus* and *Veillonella* in the salivary microbiota were positively associated with *Veillonella* and *Streptococcus* belonging to the gut microbiota. Strong positive correlations were also found between *Granulicatella* and *Corynebacterium* (saliva) with *Granulicatella* and *Corynebacterium* (stool), respectively. The positive correlation between *Gemella* (saliva) and *Gemella* (stool) was weak and probably due to its increase in the stool and to a parallel decrease in the salivary microbiota. Moreover, *Gemella* (saliva) positively interacted with *Granulicatella* (stool). The corresponding co-occurrence network showed that *Streptococcus*, *Veillonella*, *Gemella*, *Haemophilus*, and several OTUs belonging to Enterobacteriaceae that were present in stool and saliva interacted with each other within a dense cluster of positive and negative correlations (Figure 8D).

On day 180, positive correlations between *Granulicatella* and *Staphylococcus* (saliva versus stool) and negative correlations between *Streptococcus*, *Veillonella*, and *Haemophilus* (saliva versus stool) were observed. Furthermore, *Streptococcus* and *Veillonella* that were found in saliva continued to present positive correlations with *Veillonella* and *Streptococcus* that were observed in the gut microbiota, respectively (Figure 8E).

## 4. Discussion

Microbiota colonization dynamics in infancy is a topic of huge interest in the field of microbial ecology and human health [33]. In this scenario, very little is known about the development of the infant oral microbial ecosystem [34].

Our study suffers from the limited number of enrolled subjects. In our previous experiences, the recruitment of healthy subjects was more difficult with respect to the recruitment of diseased subjects. This could probably be due to the lower interest of babies’ parents to participate in research projects in the absence of a disease for which the research could be important. Moreover, since the post partum period is very stressful for parents; participation in a research project that involves the collection of samples and medical examinations can worse this situation, with the consequence of the subjects’ drop-out increasing or the negation of informed consent.

Nevertheless, this study offers a piece of knowledge on how the oral microbiota may affect the gut microbiota in healthy newborns. Particularly, the novelty of this work is the investigation of the salivary microbiota dynamics at early time points after birth as 7 and 15 days and its correlation with the development of the gut microbiota.

Interestingly, we found a core oral microbiota that was formed mainly by *Streptococcus* and *Staphylococcus* (approximately 90% of the total oral microbiota) and, unlike the gut microbiota dynamics, it was more stable during the first six months of an infants’ life. *Streptococcus* appeared to rapidly dominate the oral microbiota ecosystem and to persist during all the time course [26,35,36,37,38]. During this period, *Streptococcus* intertwined a dense web of only negative correlations with other oral bacteria, indicating its competition with other species for colonization purposes. *Streptococcus* has been demonstrated to be one of the prevalent bacterial genus’ of the breast milk ecosystem [39,40,41,42] and particularly *Streptococcus salivarius* has been frequently found in the oral cavity of breastfed infants and associated with the first oligosaccharide stimuli [43]. Furthermore, breast milk creates an immunoglobulin A1 (IgA1)-rich ecological niche in the newborn oral cavity allowing a rapid colonization of *Streptococcus* spp. capable to cleavage IgA1 [44].

A second dominant OTU that was observed in the salivary microbiota was *Staphylococcus*, previously found to be part of the milk ecosystem [36]. Thus, similarly to *Streptococcus*, it could be possibly acquired through breastfeeding from milk or mother skin contact [45,46]. Moreover, both *Streptococcus* and *Staphylococcus* are oxygen-tolerant [47] and this may explain their presence as early commensals in the salivary microbiota. The production and excretion of metabolic products of these pioneers promote a change of the oral environment that we observed during our time course, thus favoring the growth and selection of other bacteria, including more strictly anaerobes [48], as proposed for the gut microbiota [49]. In particular, *Veillonella* showed an increase of its relative abundance at 180 days’ time point together with *Haemophilus*, *Porphyromonas*, *Prevotella*, *Fusobacterium*, and *Granulicatella*, indicating that only at this stage is the environment suitable for their establishment in the oral cavity. Streptococcus’s relative abundance dropped down from 75.8% to 67% in this new niche. Its ability to inhibit the growth of other bacterial species underwent a turnaround behaviour since an increase of oral *Veillonella*, *Haemophilus*, *Porphyromonas*, *Prevotella*, *Fusobacterium*, and *Granulicatella* was found. The more reduced oral environment could explain the reason why *Veillonella*, which is continuously transferred from human milk to the neonate’s oral cavity during breastfeeding, starts to colonize infants’ mouth only after six months of age [50,51]. Regarding *Haemophilus*, *Porphyromonas*, *Prevotella*, *Fusobacterium*, and *Granulicatella*, they were all linked to developing a more mature oral microbiome [26,35]. Mainly, *Granulicatella* is considered a common dental plaque inhabitant, and, in agreement with other studies which followed the development of infant oral microbiota, an increase of its abundance was observed after six months of age [47,52]. It was hypothesized that this increment was due to teeth eruption when new ecological niches are created during six to eight months of age, promoting its colonization [26].

Analyzing the gut ecosystems’ kinetics, we found the presence of bacterial communities also in meconium, thus corroborating the hypothesis that microbial exposure may start before delivery with early bacteria pioneers that are derived from the maternal microbiota [4,7,15]. Notably, the meconium ecosystem was characterized by the dominance of Enterobacteriaceae and *Bacteroides* that were probably derived from the exposure of the newborn to mothers’ microbiota during delivery [53]. Moreover, a core gut microbiota was characterized by an in-depth analysis of the gut microbiota composition during the entire time course. This core constituted a stable ecological niche that is important for establishing the future microbiota. The other intestinal transient colonizers appeared at different time points and were influenced mainly by external (e.g., feeding and environment) and internal factors (e.g., gut maturation, gut oxygen levels, and infants’ aging), but also by the presence of bacteriophages, which can grant metabolic, immune, and evolutionary advantages to bacterial hosts [15,54].

Particularly, we found the dominance of Enterobacteriaceae and *Staphylococcus* during the first week of life, followed by *Streptococcus* colonization, suggesting that the facultative anaerobes are the first colonizers in the human gut. They provide a reduced environment that is favorable for the establishment of later-occurring anaerobic bacteria such as *Bifidobacterium*, *Eggerthella*, *Bacteroides*, and *Veillonella* [53,55]. As assessed with the oral microbiota, in the process of transition from an oxygen-tolerant to a more anaerobic gut microbiota, a possible interaction between *Streptococcus* spp. and *Veillonella*, towards the last time points of the time course, could be established. Once it reaches the gut, the human milk (7% lactose and 1% human milk oligosaccharides [HMOs]) becomes the substrate of several bacteria such as *Streptococcus*, which is involved in human milk fermentation transforming lactose into lactate [56,57]. At the same time, *Veillonella* may be able to utilize the formed lactate to produce propionate in a cross-feeding phenomenon [58,59,60]. The more reduced environment and the presence of HMOs induce the colonization of the gut microbiota by *Bifidobacterium*, which is a characteristic colonizer of human milk and is important for inhibiting the growth of pathogenic microorganisms, modulating mucosal barrier function, and promoting immunological and inflammatory responses [36,61].

Gut microbiota heatmaps and co-occurrence networks revealed that, up to 15 days, *Bifidobacterium* correlated positively with oral- or breastfeed-belonging bacteria such as *Staphylococcus*, *Streptococcus*, *Lactobacillus*, *Gemella*, and *Haemophilus.* Moreover, in the following time points, when the gut ecosystem starts to become more anaerobic, new positive and negative correlations took place with *Veillonella*, *Bacteroides*, *Clostridium*, and several members of Proteobacteria that are associated with a more mature gut microbiota [17,49]. Thus, confirming that, in the development of infants’ physiological microbiota, environmental conditions, such as the pH, oxygen levels, and nutrients availability, influence the microbial species selection and vice versa in a cascade of time-dependent events.

The mouth represents the main route that is followed by bacteria to reach the gastrointestinal tract. The influence of the oral microbiota on the shape of the gut microbiota is described in a few studies. In adults, these two communities have low overlap, while in infants the role of the oral-gut axis in the gut microbiota development is undoubtedly important [36,62,63].

Moreover, longitudinal studies that focused on the simultaneous analysis of oral and intestinal microbiota development with culture-independent next-generation sequencing methodologies are very scarce and limited to only taxa description without any network and correlation implication between the communities [62,64].

Then, in our study we applied these approaches to a human observational study to reveal the influence of the two salivary and fecal ecosystems during infancy. Despite the difficulty of discriminating between genuine and spurious correlations, these approaches have been applied in a gnotobiotic mouse model to understand the ecological invasion of salivary bacteria on the gut [35].

In our study, ecological analyses revealed that both the composition and the structure of the two microbiota ecosystems, evolve as infants’ grow. Thus, the maturation of the microbiota is a nonrandom process, but it is driven by specific interactions between the taxa that change during the time course of the programming [17,36,65] and the niche environment is the primary driver of the composition of the local microbiome, as already described [66].

Interestingly, the salivary microbiota showed an increase in the total correlations from 15 to 30 days, probably due to the milk maturation. Indeed, the number of interactions among the OTUs may increase proportionally with the higher intake of nutrients, linked not only to breast milk composition but also to the increased volume of the milk intake [53,59].

By our results, we assume that the oral and gut microbiota share, during the entire time course, three seminal OTUs, namely *Staphylococcus*, *Streptococcus*, and *Veillonella* that could be able to colonize the gut microbiota in a differential way. Moreover, *Staphylococcus* was stable in the salivary microbiota during all time course, while in the gut decreased over time, probably because of the development of a more anaerobic environment. At seven days, when *Staphylococcus* reached a very high concentration in the oral and gut microbiota, a dense hub of connections became established with other bacteria in the stools. During the remaining time points, the oral and gut *Staphylococcus* presented a very low number of connections (maximum three) with the gut and oral microbiota members. Thus, oral *Staphylococcus* seemed to not influence the shape of gut microbiota. On the other side, *Streptococcus* and *Veillonella* established a cooperative interaction during the last points of the time course in both the oral and gut ecosystems. These results support the hypothesis that not only external and/or internal factors (i.e., environmental, feeding mode, oxygen level, age) are involved in the development of the specialized oral and gut microbiota, but also some specific oral OTUs may influence the gut microbiota modeling processes as seeding species.

## 5. Conclusions

In conclusion, the findings from our small but homogeneous cohort continue to enlarge the knowledge on the progressive construction of infants’ microbiota. From an ecological point of view, our results suggest that the process of microbiota assembly is driven principally by local adaptation to the environmental niche. Moreover, we assume a possible influence of some oral bacteria in the process of gut microbiota development.

However, further studies at the species and strain level are needed to confirm a direct transmission; hence, shotgun sequencing approaches should be recommended to track the bacteria that are moving from a district to another one. The deep knowledge of how oral microbiota intervenes in the modeling of the gut microbiota in healthy infancy may open new avenues on the design of post-biotics. In fact, an ad hoc designed post-biotics that is based on the shared oral- and gut-targeting microbes could improve dysbiotic condition of gut microbiota, especially targeting potential pathogens of the entire gastro-intestinal tract or restoring deprived communities by introducing eubiotic bacteria. These new therapies could be applied in premature birth neonates or in the presence of early life intestinal diseases (e.g., Hirschsprung’s disease or intestinal ischemia) in which gut dysbiosis plays a key role in worsening the health conditions.

## Figures and Tables

**Table 1:** The general characteristics of the neonate cohort.

Characteristics	Time Point					
Age (days)	0	7	15	30	90	180
Subjects (no.)	5	19	15	18	15	15
Saliva samples	0	19	15	18	15	15
Stool samples	5	17	14	15	14	15
Gestational age (mean, weeks)	39.2	39.4	39.4	39.3	39.3	39.3
Female/male	1/4	9/10	5/10	10/8	8/7	9/6
Birth weight (g) mean ± SEM ^a^	3528 ± 95	3418 ± 75	3506 ± 102	3267 ± 69	3350 ± 58	3221 ± 81
Weight at the time of stool/saliva collection (g) mean ± SEM ^a^	3430 ± 100	3390 ± 82	3744 ± 76	4099 ± 118	5828 ± 134	6979 ± 172
Weaning ^b^	No	No	No	No	No	Yes (10/15)

^a^ SEM, standard error of the mean; ^b^ at 180 days 10/15 infants started weaning mostly with fruit (mainly apple and pear).

## Data Availability

All raw sequences have been archived in the NCBI database: PRJNA753008 (https://www.ncbi.nlm.nih.gov/bioproject, accessed on 11 January 2022).

## Supplementary Materials

The following supporting information can be downloaded at: https://www.mdpi.com/article/10.3390/microorganisms10020468/s1, Supplementary Table S1. Wilcoxon test between the saliva and stool α-diversity indices at each time point of follow-up. Supplementary Table S2. The average of relative abundances of the OTUs that are shared in the saliva samples (core saliva microbiota) in all time-course points. Supplementary Table S3. Average of the relative abundances of the OTUs that are shared in the fecal samples (core gut microbiota) in all points of the time-course. Supplementary Table S4. OTUs that are shared between the gut and salivary microbiota of the cohort of infants at each point of the time course. Supplementary Table S5. Characteristics of networks of the saliva, stool, and saliva versus the stool samples. Supplementary Figure S1. Principal component analysis (PCA) plots applied on the OTUs abundances of the stool and salivary microbiota of all the children of the study considering gender variable. M, male; F, female. Supplementary Figure S2. Principal coordinates analysis (PCoA) plots of unweighted UniFrac matrix of all time-points for saliva and stool samples. The shape of the symbols refers to the microbiota matrix, while the color indicates the time point. Red and blue ellipses highlight saliva and stool samples, respectively. The plots show the first two principal axes for PcoA using the unweighted UniFrac algorithm. Supplementary Figure S3. Colonization trends of the bacterial genera shared in all points of the time course between salivary (A) and gut (B) ecosystems.
