# Supplementary material for: A Parallel Tracking of Salivary and Gut Microbiota Profiles Can Reveal Maturation and Interplay of Early Life Microbial Communities in Healthy Infants
Source: Microorganisms. 2022 Feb 18;10(2):468. doi: 10.3390/microorganisms10020468 (PMC8880349; doi:10.3390/microorganisms10020468)
Supplement: Supplementary file 1 [file microorganisms-10-00468-s001.zip › microorganisms-1573986-supplementary-revised.pdf]

Supplementary Materials

Supplementary Tables:

Supplementary Table S1. Wilcoxon test between saliva and stool α-diversity indices at each time point of follow-up.

Alpha diversity indexes	time point	Saliva median	Stool median	FDR p value
Chao1	7 days	36.50	14.00	0.0078
	15 days	76.00	19.50	0.0002
	30 days	47.63	26.00	0.0238
	90 days	43.20	19.50	0.0164
	180 days	55.20	17.00	0.0005
Shannon	7 days	1.88	1.26	0.0112
	15 days	2.82	1.51	0.0028
	30 days	2.13	1.98	0.1533
	90 days	2.20	1.47	0.0195
	180 days	2.62	1.64	0.0019

Supplementary Table S2. Average of relative abundances of OTUs shared in saliva samples (core saliva microbiota) in all time-course points.

Genus	7 days	15 days	30 days	90 days	180 days
Rothia	0.013	0.010	0.018	1.615	0.217
Staphylococcus	17.630	18.948	15.391	16.996	12.791
Streptococcus	71.308	77.453	79.661	75.874	66.999
Gemellaceae_g	6.494	1.008	0.714	0.944	2.322
Veillonella	0.006	0.041	0.098	1.583	4.976

Supplementary Table S3. Average of relative abundances of OTUs shared in faecal samples (core gut microbiota) in all points of the time-course.

**Genus**	**BIRTH**	**7 days**	**15 days**	**30 days**	**90 days**	**180 days**
Enterobacteriaceae_g	81.186	13.110	21.255	26.289	14.122	14.749
Erwinia	0.012	0.022	0.141	0.277	0.034	0.176
Klebsiella	0.024	0.011	0.035	0.027	0.023	0.181
Bacteroides	3.458	4.180	13.095	8.197	1.295	0.191
Parabacteroides	0.002	0.001	0.012	0.222	0.007	0.003
Bifidobacterium	0.142	0.013	2.271	6.935	41.467	13.901
Akkermansia	0.001	0.002	0.023	0.183	0.009	0.106
Staphylococcus	0.002	4.031	1.249	0.153	0.350	0.031
Enterococcus	0.001	0.038	0.015	0.121	0.643	0.607
Streptococcus	0.067	1.592	0.520	1.455	4.068	3.051
Ruminococcaceae_g	0.105	0.005	0.012	1.405	0.788	0.146
Lachnospiraceae_g	0.087	0.005	0.036	0.118	0.114	0.059
Clostridium	0.001	0.002	0.004	0.005	0.008	0.010
Veillonella	0.347	0.022	0.378	0.175	0.301	1.528

Supplementary Table S4. OTUs shared between the gut and salivary microbiota of the cohort of infants at each point of the time course.

Shared OTUs[a]				
**7 days**	**15 days**	**30 days**	**90 days**	**180 days**
Streptococcus	***Streptococcus***	***Streptococcus***	***Streptococcus***	***Streptococcus***
Staphylococcus	***Staphylococcus***	***Staphylococcus***	***Staphylococcus***	***Staphylococcus***
Veillonella	***Veillonella***	***Veillonella***	***Veillonella***	***Veillonella***
Haemophilus	Planococcaceae_g	Enterobacteriaceae_g	Enterobacteriaceae_g	*Gemella*
Acinetobacter	Enterobacteriaceae_g		*Corynebacterium*	Planococcaceae_g
Gemella	Gemellaceae_g		*Haemophilus*	Gemellaceae_g
Planococcaceae_g			Gemellaceae_g	*Granulicatella*
Rothia				*Haemophilus*
Gemellaceae_g				*Prevotella*

[a] in bold are highlighted OTUs shared between gut and salivary microbiota in all points of the time course.

Supplementary Table S5. Characteristics of networks of saliva, stool and saliva versus stool samples.

	Saliva					***Stool***						***Stool vs. Saliva***				
	7 days	***15 days***	***30 days***	***90 days***	***180 days***	***Birth***	***7 days***	***15 days***	***30 days***	***90 days***	***180 days***	***7 days***	***15 days***	***30 days***	***90 days***	***180 days***
Avg. number of neighbors	5.48	5.95	9.13	9.10	10.81	14.03	12.11	11.55	8.82	12.86	8.06	5.51	5.40	8.27	5.53	6.58
Network diameter	12	5	8	10	8	8	8	8	11	9	11	6	6	9	8	8
Characteristic path length	3.12	1.96	3.17	4.13	3.00	3.50	3.97	2.79	4.34	3.26	4.71	2.08	2.27	3.96	3.21	3.45
Clustering coefficient	0.73	0.77	0.84	0.83	0.79	0.94	0.90	0.92	0.83	0.87	0.85	0.00	0.00	0.00	0.00	0.00
Network centralization	0.16	0.21	0.19	0.16	0.15	0.11	0.11	0.14	0.11	0.24	0.10	0.06	0.05	0.09	0.07	0.07
Network heterogeneity	0.67	0.54	0.48	0.51	0.61	0.41	0.51	0.71	0.43	0.70	0.46	0.65	0.40	0.51	0.57	0.55
Network radius	1	1	1	5	1	1	1	1	1	1	1	1	1	1	1	1
Network density	0.11	0.15	0.14	0.16	0.14	0.19	0.10	0.11	0.08	0.12	0.08	0.07	0.09	0.09	0.07	0.07
Connected components	6	5	2	1	3	4	2	7	3	4	6	11	6	3	7	5
Shortest paths	722	594	3666	3306	4984	2346	1217	2660	9714	9202	5648	1230	996	8042	2228	6080
Number of nodes	50	41	64	58	77	75	124	102	105	112	107	78	60	98	76	96

Supplementary Figures

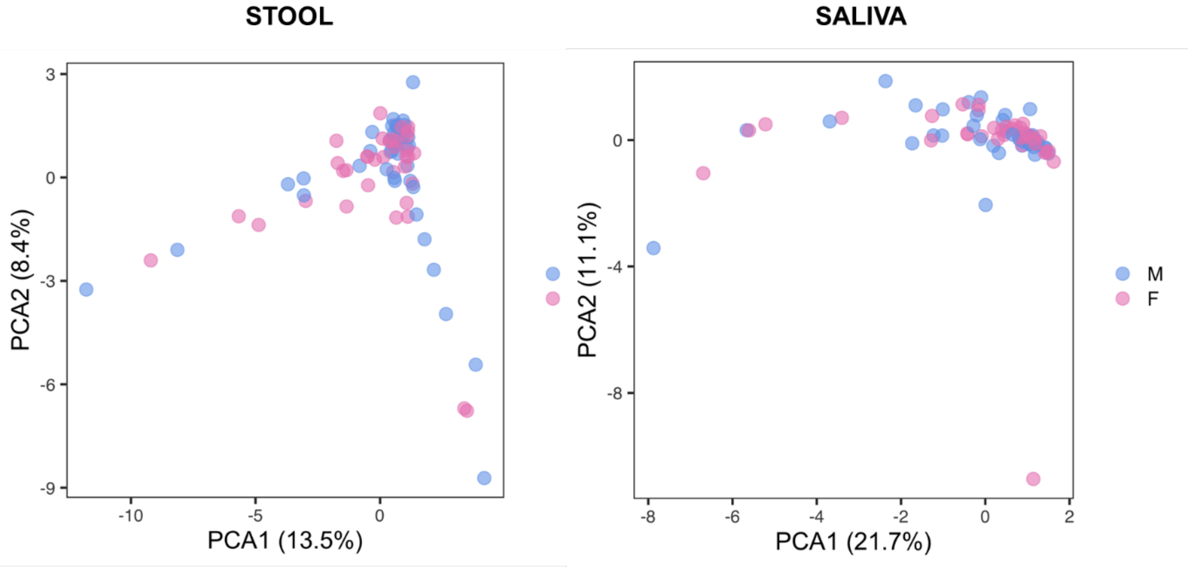

Supplementary Figure S1. Principal Component Analysis (PCA) plots applied on OTUs abundances of stool and salivary microbiota of all the children of the study considering gender variable. M, male; F, female.

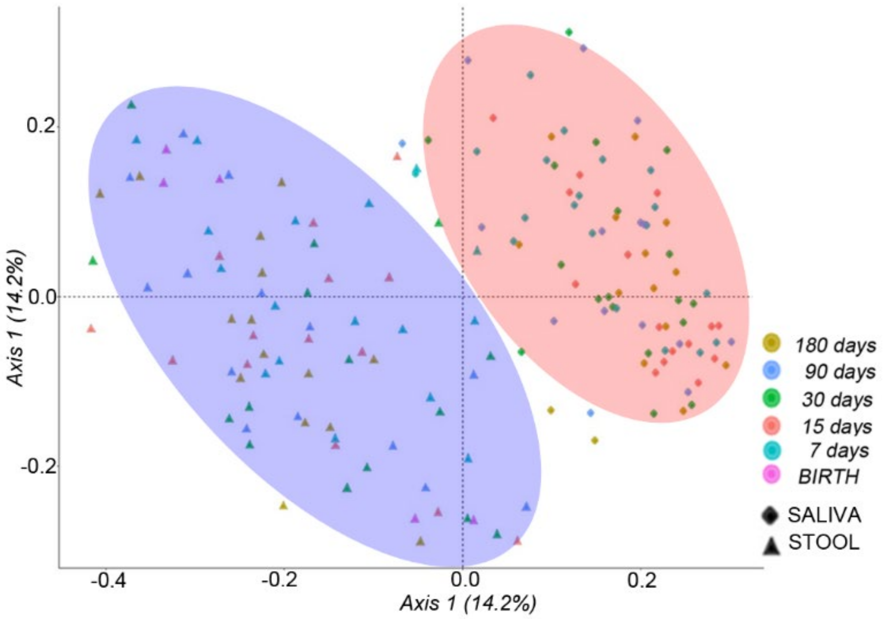

Supplementary Figure S2. Principal Coordinates Analysis (PCoA) plots of unweighted UniFrac matrix of all time-points for saliva and stool samples**.** The shape of the symbols refers to the microbiota matrix, while the color indicates the time point. Red and blue ellipses highlight saliva and stool samples, respectively. The plots show the first two principal axes for PCoA using the unweighted UniFrac algorithm.

A B

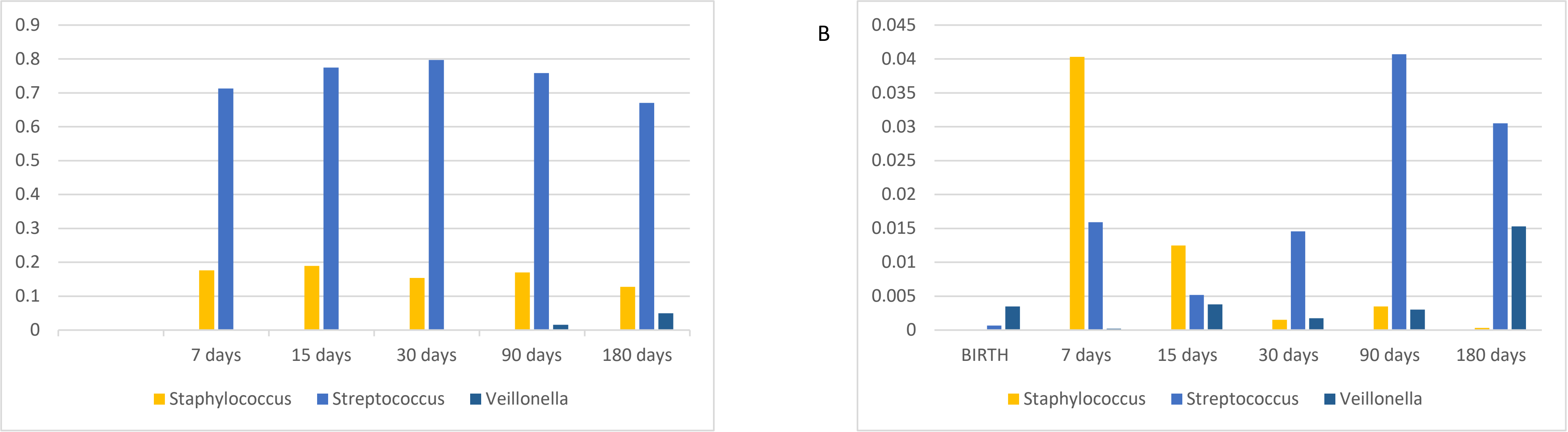

Supplementary Figure S3. Colonization trends of the bacterial genera shared in all points of the time course between salivary (A) and gut (B) ecosystems.
